# Mental health in children with living donor liver transplantation: a propensity score-matched analysis

**DOI:** 10.1186/s13034-022-00516-4

**Published:** 2022-11-29

**Authors:** Mingzhu Huang, Yuchen Hou, Wen Li, Guanghai Wang, Guangxiang Gu, Qiang Xia

**Affiliations:** 1 Department of Liver Surgery, Renji Hospital, School of Medicine, Shanghai Jiao Tong University, No.160 Pujian Road, Pudong New District, 200128 Shanghai, China; 2 Department of General Surgery, the First Affiliated Hospital of USTC, Division of Life Sciences and Medicine, University of Science and Technology of China, 230021 Hefei, China; 3 Department of Pediatrics, The First Affiliated Hospital of Zhengzhou University, No.1 Jianshe Dong Road, 400052 Zhengzhou, China; 4 Pediatric Translational Medicine Institution, Department of Developmental and Behavioral Pediatrics, Shanghai Children’s Medical Center, School of Medicine, Shanghai Jiao Tong University, 200127 Shanghai, China; 5 MOE-Shanghai Key Laboratory of Children’s Environmental Health, School of Medicine, Xinhua Hospital, Shanghai Jiao Tong University, 200092, Shanghai, China; 6 Shanghai Center for Brain Science and Brain-Inspired Technology, 201602, Shanghai, China; 7 Department of Liver Transplantation, Sun Yet-sen Memorial Hospital, Sun Yat-sen University, No.107 Yanjiang West Road, 510080, Guangzhou, China

**Keywords:** Pediatric liver transplantation, Mental health, Lifestyle and family environment factors, Retrospective study

## Abstract

**Background:**

This study explored mental health of pediatric patients with living donor liver transplantation.

**Methods:**

A total of 741 children who successfully underwent living donor liver transplantation from 2009 to 2019 enrolled in this study. Participants were aged between 3 and 12 years (mean age = 5.28; SD = 2.01). The Strengths and Difficulties Questionnaire was used to evaluate emotional and behavioral problems. Parents completed the 5-item World Health Organization Well-Being Index and reported their child’s height, weight, sleep duration, parent-child interactions, home environment, physical activities, and time spent on screen exposure. Propensity score matching method was used to generate a control group from 20,934 healthy children. Univariate analysis and multiple logistic regression analyses were used to identify the correlational factors in children’s mental health following a liver transplantation.

**Results:**

Compared to healthy children, patients after liver transplantation were prone to emotional problems, hyperactivity, and peer problems. Moreover, parental mental health, physical activity, and family environment were identified as factors associated with mental health of pediatric liver transplant patients.

**Conclusion:**

The findings highlight the need to focus on mental health of pediatric transplant patients, increase support for parents, and strengthen positive parent-child interactions.

**Supplementary Information:**

The online version contains supplementary material available at 10.1186/s13034-022-00516-4.

## Background

Liver transplantation has become a standard treatment for acute liver failure or progressed end-stage liver disease caused by pediatric liver diseases [1]. Novel, continuously emerging therapies, and immunosuppressive agents for liver transplantation have improved the quality of life (QOL) of transplant recipients over the past decades. For example, the 1-year survival rate for pediatric liver transplant patients is over 90%, and the 5-year survival rate is over 85% [2, 3]. However, despite these advantages, previous studies have indicated that surgery can reduce children’s social interactions, leading to impaired psychosocial wellbeing in 17.7% of pediatric patients [4, 5]. Social deprivation in adolescence has been indicated in neurochemistry, structural brain development, and behaviors associated with mental health problems [6, 7]. This indicates that post-surgery pediatric patients may be vulnerable to mental health problems. Therefore, consideration of QOL, particularly regarding children’s mental health, is essential in medical decision-making.

Compared to a physically healthy population, children with a history of undergoing surgery are vulnerable to unhealthy lifestyle habits, such as lower level of physical activity. Furthermore, parents’ socioeconomic status and mental health tend to decline after children’s surgery, causing children to experience the adverse effects of parental stress [8, 9]. Extant literature in this area has demonstrated that these factors are associated with an increased risk of poor mental health. For example, insufficient physical activity is inversely linked to mental health problems in children [10]. Additionally, parental mental health problems, particularly maternal depression, can also affect the psychological health of young children [11]. We found an increased risk of mental health problems among adolescent and young adult recipients of liver transplants. However, there is a lack of resources for conducting studies among this population, as well as a paucity of research targeting younger children, which is a crucial, sensitive period in life to promote emotional wellbeing and to ensure long-term mental health. Previous research has confirmed that the mental health of young children can predict their long-term psychological development [12]. Furthermore, there is no direct and scientific evidence regarding the association of lifestyle and home environment factors with children’s mental health after liver transplantation in China and other countries. Such information would prove indispensable for monitoring and preventing the detrimental effects of surgery on children’s mental health.

Therefore, we conducted a population-based online survey of physically healthy children and children who underwent transplant surgery. The study aimed to elucidate the effects of liver transplantation on children’s mental health, and further explored the associated factors of lifestyle and family environment.

To our knowledge, this first large-sample and retrospective study would provide important insights for improving the mental health and long-term outcomes of children after living donor liver transplantation.

## Methods

### Participants

Between January 2009 and December 2019, a total of 741 children aged between 3 and 12 years successfully underwent living donor liver transplantation at the Department of Liver Surgery, Renji Hospital, School of Medicine, Shanghai Jiaotong Universit y. Children were identified at the time of surgery, and the median follow-up time was 42.07 (range = 2.77–131.97) months. All pediatric liver transplantation procedures were approved by the ethics committee and were performed according to the relevant regulations. Children aged between 3 and 12 years were eligible for participation in the study. Those who experienced postoperative surgical complications or re-transplantation were excluded. This study was approved by the Ethics Committee of Renji Hospital, School of Medicine, Shanghai Jiao Tong University, and was conducted in accordance with the declarations of Helsinki and Istanbul. Informed consent was obtained from all participants.

Control data were obtained from a populational survey of physically healthy children aged between 3 and 12 years in mainland China. The survey was conducted from March 15th to 29th, 2020. It was distributed via the widely used We-Chat based online survey platform, Questionnaire Star (https://www.wjx.cn/).

### Children’s mental health

The Strengths and Difficulties Questionnaire (SDQ) is one of the most widely used tools to identify mental health problems in children and adolescents aged 3 to 16 years [13]. This parent-reported questionnaire was used to assess the psychosocial well-being of children (emotional symptoms, conduct problems, hyperactivity/inattention, peer relationship problems, and prosocial behavior). The first four subscales (excluding prosocial behavior) were combined to generate a total difficulties score, which was used as a binary variable in the analysis. A total score of ≥ 14 indicated emotional and behavioral problems, and a score of ≥ 6 indicated prosocial behavior [14].

### Parental mental health

The 5-Item World Health Organization Well-being Index (WHO-5) is globally used to assess subjective psychological well-being. It was derived from the World Health Organization Ten Well-being Index [15]. Participants rated the degree to which they experienced positive emotions in the preceding two weeks. The answers were reported on a 6-point Likert-type scale, ranging from 0 (“never”) to 5 (“always”). A score of ≤ 13 is indicative of depression [16]. In adults, the WHO-5 has proven to be an extremely sensitive screening tool for detecting depressive affect.

### Parent-child interaction

Parent-child interaction was assessed using the Chinese Parent-Child Interaction Scale (CPCIS). This standardized questionnaire has been well-validated in China [17]. The questionnaire measures how frequently participants engaged in 12 common parent-child activities during the past month. Activities include learning-related tasks, reading, recreation, and interaction with the environment, among others. Answers were recorded on a 6-point Likert-type scale ranging from 0 (“not occurring”) to 5 (“very frequently occurring”). The total scores of all activities were used in the analysis. Higher scores indicated more frequent parent-child interactive activities.

### Socioeconomic status

Parents reported maternal education and annual household income. Maternal educational qualification was categorized into three groups: senior high school or lower, graduate, and post-graduate. Annual household income was divided into five categories: <30,000 RMB (Renminbi); 30,000–50,000 RMB; 50,000-100,000 RMB; ≥100 000 RMB; and “unknown”.

### General home environment

The Index of Child Care Environment (ICCE) was used to assess the children’s care environment. The ICCE consists of 13 questions in 4 subscales: human stimulation, social stimulation, avoidance of restriction, and social support. Each item was assessed using a multiple-choice question format, and the answer was given a binary score according to the manual (1 = “good”; 0 = “not good” or “not sure”). The overall score was calculated by summing the scores of all items in the scale. A higher score indicated a better childcare environment.

### Statistical analyses

The propensity score matching (PSM) method was used to generate a control group to facilitate comparability [18]; specifically, the nearest neighbor and optimal matching methods are the most popular matching approaches used by applied scientists. Using these approaches, one individual from the treatment group was matched with one or more individuals from the control group (1:2 in this study) if their propensity scores were within a pre-specified range. The density and distribution of propensity scores are shown in Supplementary Fig. 1 and Fig. 2 (see Additional file 1). Descriptive analysis was conducted to compare the socio-demographic variables of the post-surgery and control groups. Student’s t-test and Chi-square test were used to compare children’s mental health status with activities of varying intensities, parental mental health, bed-time screen exposure time, ICCE, and CPCIS scores. Logistic regression analysis was performed to explore the association between children’s mental health and physical activity, parental mental health, and parent-child interactions. Effect sizes were presented as odds risks (ORs) with 95% confidence intervals (CIs).

PSM was performed using the package MatchIt in R (version 4.0.2) and the ratio was set at 1:2. Descriptive and regression analyses were conducted using Stata v 14.2 (StataCorp LP, College Station, Texas).

## Results

As detailed in Table 1, there were more women in the post-surgery group (post-surgery group vs. control group: 53.98% vs. 46.02%; *p* = 0.002). Children in the post-surgery group exhibited more emotional and behavioral problems (post-surgery group vs. control group: 46.02% vs. 32.20%; *p* < 0.001) and tended to have mothers with more mental health problems (post-surgery group vs. control group: 38.06% vs. 25.36%; *p* < 0.001). Chi-square analyses were performed to compare the prevalence of various aspects of child mental health using SDQ scores between the different groups (Table 2).

**Table 1:** Socio-demographic characters of children from the liver transplantation and control groups

Characteristics	Post-surgery group (n = 741)	Control group (n = 1471)	*P* value
**Gender**, N(%)			0.002
Male	341 (46.02)	782 (53.2)	
Female	400 (53.98)	689 (46.8)	
**Age, median (IQR), y**	5.28 (2.06, 7.38)	5.30 (1.31,7.50)	0.745
**Age of liver transplantation, median (IQR), y**	0.75(0.58,2.58)	NA	
**Years of follow-up, median (IQR)**	3.30(2.55,4.73)	NA	
**Siblings**, N(%)			0.962
None	271 (36.57)	541 (36.78)	
One or more	470 (63.43)	930 (63.22)	
**Family Income (RMB)**, N(%)			0.982
30 K or less	161 (21.73)	312 (21.21)	
30-50 K	170 (22.94)	340 (23.11)	
50-100 K	154 (20.78)	308 (20.94)	
100 K or more	183 (24.69)	355 (24.13)	
**Parental education**, N(%)			0.198
Senior high school or below	234 (31.58)	468 (31.82)	
Undergraduate degree	201 (27.13)	401 (27.26)	
Master’s degree or above	306 (41.30)	593 (12.63)	
**Parental mental health (WHO-5)**, N(%)			< 0.001
Normal	459 (61.94)	1098 (74.63)	
Abnormal	282 (38.06)	373 (25.36)	

**Table 2:** Prevalence of emotional/behavioral problems in children after liver transplantation and the control group

	Cut-off	Post-surgery group			Control group		P value
		**M (SD)**	**Prevalence**		**M (SD)**	**Prevalence**	
**SDQ**							
**Total score**	13	13.57 (5.29)	46.02		11.90 (5.12)	32.20	< 0.0001
**Hyperactivity**	5	5.32 (2.28)	45.61		4.48 (2.12)	30.08	< 0.0001
**Peer problems**	2	2.93 (1.68)	58.03		3.04 (1.52)	61.37	0.129
**Conduct**	2	2.58 (1.57)	46.15		2.14 (1.55)	31.55	< 0.0001
**Emotional problems**	3	2.75 (2.11)	32.52		2.24 (1.96)	22.08	< 0.0001
**Prosocial behavior**	5	6.81 (2.27)	29.42		6.76 (2.02)	29.97	0.806

**Fig. 1:**
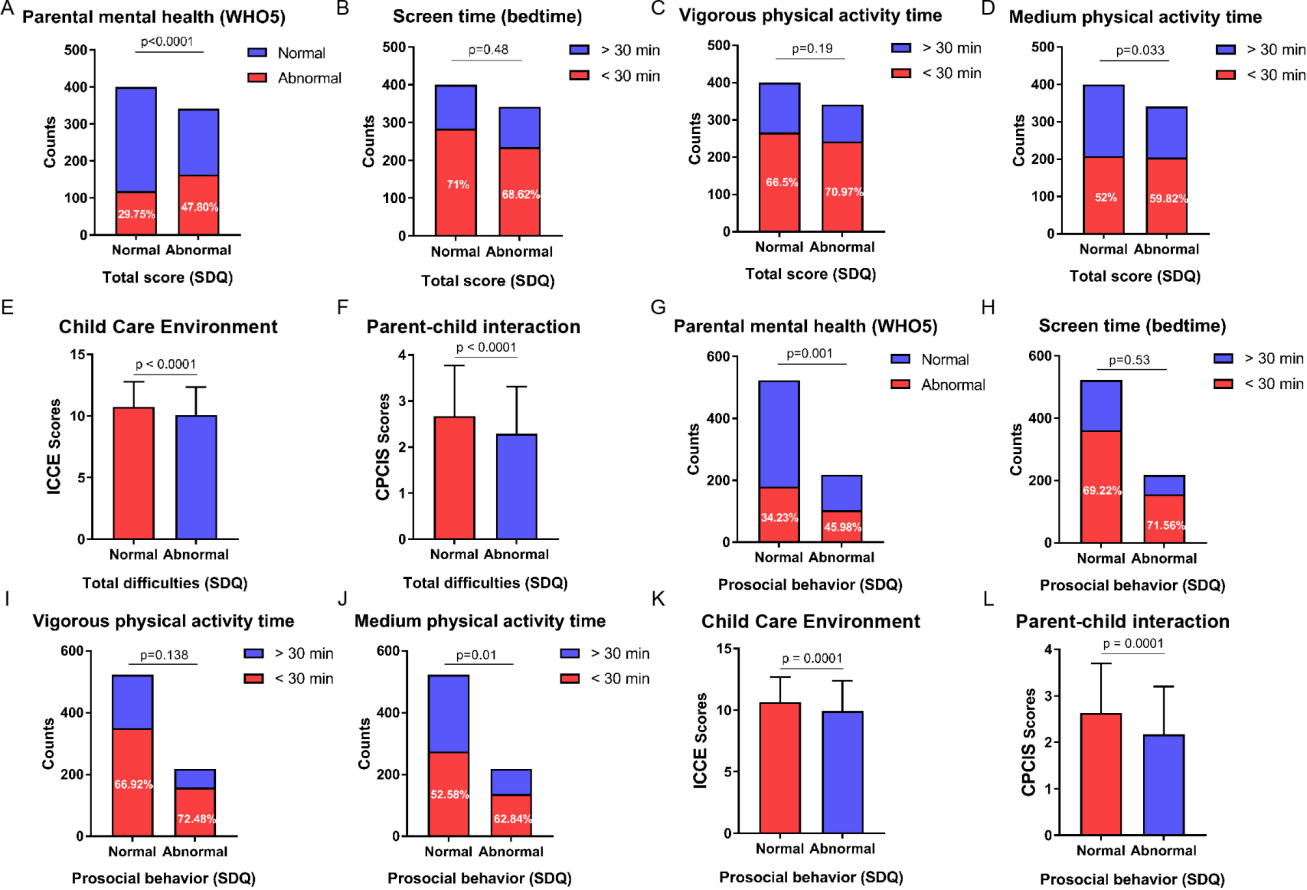
Prevalence of emotional and behavioral problems in the post-surgery group. For pediatric transplant patients, abnormal parental mental health, medium-intensity physical activity duration, poor child-care environment, and inadequate parent-child interactions were associated with a higher prevalence of emotional and behavioral problems

Chi-square testing was used to compare the prevalence of child mental health problems in the post-surgery group according to lifestyle and home environment factors. The prevalence of mental health problems was higher among children with parental mental health problems (*p* < 0.001), those engaging in insufficient physical activity (*p* = 0.033), and those with lower ICCE (*p* < 0.001) and CPCIS scores *(p* < 0.001). Meanwhile, children’s prosocial behaviors were also correlated with parental mental health (p = 0.001), duration of medium-intensity physical activity (*p* = 0.01), ICCE scores (*p* = 0.001), and CPCIS scores (*p* = 0.001), as indicated in Fig. 1.

Logistic regression model analyses indicated that engaging in medium-intensity physical activity for over 30 min per day and high-quality parent-child interactions were protective factors for the mental health of children post-surgery. The adjusted ORs of medium-intensity physical activity for over 30 min per day and high-quality parent-child interactions to emotional and behavioral difficulties were 0.73 (95% CI: 0.54–0.99; p = 0.044) and 0.64 (95% CI: 0.47–0.87; p = 0.004), respectively. Additionally, parental mental problems posed a risk factor, with an adjusted OR of 2.17 (95% CI: 1.59–2.97; p < 0.0001). Children who engaged in physical activity for over 30 min per day (adjusted OR 0.61; 95% CI: 0.44–0.86; p = 0.004) and had good CPCIS scores (adjusted OR 0.56; 95% CI: 0.42–0.79; p = 0.001) were likely to exhibit prosocial behaviors. However, parental mental health problems (adjusted OR 1.66; 95% CI: 1.20–2.31; p = 0.003) was an independent risk factor (Table 3). The results were adjusted according to age, sex, family income, and parental education.

**Table 3:** Associated factors affecting the mental health of pediatric liver transplant recipients

	Total score (SDQ)						Prosocial behaviors				
	Unadjusted OR (95% CI)	P value		Adjusted OR (95% CI)	P value		Unadjusted OR (95% CI)	P value		Adjusted OR (95% CI)	P value
**Medium-intensity physical activity**											
< 30 min/day	REF.	REF.		REF.	REF.		REF.	REF.		REF.	REF.
> 30 min/day	0.73 (0.54–0.97)	0.033		0.73 (0.54–0.99)	0.044		0.66 (0.47–0.91)	0.011		0.61 (0.44–0.86)	0.004
**Parental mental health (WHO-5)**											
Normal	REF.	REF.		REF.	REF.		REF.	REF.		REF.	REF.
Abnormal	2.16 (1.60–2.92)	< 0.0001		2.17 (1.59–2.97)	< 0.0001		1.72 (1.25–2.37)	0.001		1.66 (1.20–2.31)	0.003
**CPCIS**											
Bad	REF.	REF.		REF.	REF.		REF.	REF.		REF.	REF.
Good	0.55 (0.41–0.74)	< 0.0001		0.64 (0.47–0.87)	0.004		0.57 (0.42–0.79)	0.001		0.56 (0.40–0.78)	0.001

## Discussion

To our knowledge, this is the first study to assess the association between children’s mental health status after liver transplantation and its associated factors in the context of their daily lives. The findings indicate that children who received transplants were more likely to have mental health problems. Additionally, parental mental health problems, less time spent doing physical activities, and inadequate parent-child interactions were associated with higher mental health risks for children post-surgery. The findings highlight that the mental health of children who have undergone transplantation surgery should be a focal point of research. Furthermore, this study reveals the importance of parental mental health, parent-child interactions, and physical activity in promoting mental health among children who have undergone transplantation surgery. This information is pertinent to developing health programs aimed at parents of pediatric transplant patients.

Our results have provided a valid theoretical basis for improving the mental health of children after liver transplantation. With the development of pediatric liver transplantation, the focus has shifted from patient survival to a healthier QOL. Mental health problems affect these children’s daily social lives and may also impact graft survival-rates across organ transplant groups.

### Prevalence of emotional and behavioral problems in children post-surgery

Our results indicated that the prevalence of emotional and behavioral problems was significantly higher for children in the post-surgery group than in the control group. A multicenter study in North America [19] showed significantly lower Health Related Quality of Life (HRQOL) scores in children with liver transplants compared with healthy children, especially in the categories of school performance and emotional functioning. A single center follow-up in Chile [20] showed that patients under the age of four years consistently indicated good HRQOL scores. This may be because parents answered the questionnaire. Contrarily, most school-age patients reported poor HRQOL in all aspects, likely because of their irregular attendance of school activities. Another study in Germany [21] demonstrated that there was notable family strain surrounding the pediatric liver transplant recipients, when compared with a normative sample of chronically ill children, or children with disabilities. Significant associations have been observed between family strain in parents and psychosocial dysfunction in children post-surgery. These findings partly support our hypotheses, and our study confirms and extends existing research in this area. Children in the post-surgery group were more susceptible to developing psychological problems than those in the control group. Thus, maintenance of good mental health supports children’s well-being, which is an essential goal for care before, during, and post liver transplantation.

### Factors associated with the mental health of children post-surgery

Our results indicated that engaging in physical activity, quality of parent-child interactions, and parental mental health were factors associated with the mental health of pediatric liver transplantation patients. Furthermore, a lack of physical activity, inadequate parent-child interactions, and parental mental problems can serve as independent risk factors for declining mental health in pediatric liver transplantation patients.

Our results also showed that medium-intensity physical activity was associated with decreased mental health burden for pediatric transplant patients. Physical activity may improve mental health by facilitating the release of endorphins and the increase of brain-derived neurotropic factor and the growth of new capillaries, enhancing the structural and functional compositions of the brain [22]. Children with chronic illnesses are prone to having lower levels of social competence, which may negatively impact peer relationships, which is a predominant influential factor on self-esteem and general well-being [23, 24]. Physical activity improves self-concept and mental health in young people through several psychosocial paths [25]. Physical activity can also improve mental health through a range of potential behavioral mechanisms including sleep duration, sleep efficiency, sleep onset latency, and reduced sleepiness [26].

An exercise duration of ≥ 60 min was proven to significantly improve overall mental health, compared to those peers who engaged in an exercise program of < 60 min [27]. In a large U.S. sample, physical activity was reported to improve mental health burden, and an exercise duration of 45 min per day, 3‒5 times per week, was identified as the most important factor [28]. However, increased duration and intensity of exercise were not necessarily better for children. In this study, we found that children who engaged in vigorous physical activity for 30 min or more per day did not have improved mental states. This may be related to the physical health of pediatric transplant patients, and/or the results of excessive attention from caregivers.

Consistent with the literature on physically healthy children, our findings demonstrated that positive parent-child interactions improved the mental health of children who had undergone transplant surgery. A positive, nurturing environment, especially with high-quality parent-child interactions, plays a vital role in early childhood development, including cognitive and psychosocial development [29, 30]. Previous evidence suggests that high frequencies of positive parent-child interactions have positive effects on children’s psychosocial functioning [31]. Additionally, positive caregiver-child interactions can buffer the adverse effects of maltreatment on social difficulties [32].

The current research shows that up to one-third of parents of children suffering from a life-threatening illness have some symptoms of general stress disorder, such as avoidance behaviors, impulsive thoughts, or irritability [33]. However, little is known about the impact of the transplant experience and the ongoing health issues on children’s families, especially their parents. Evidently, parents play an essential role in their child(ren)’s development and they must assume a tremendous amount of responsibility [21]. This study demonstrated strong inter-relations between family strain and emotional and behavioral disturbances in children who received liver transplants. The most important factor affecting children’s ability to cope with treatment is the response of their parents to the disease and treatment [34]. Reciprocity can be assumed in that if the parents are heavily burdened and/or anxious, it may cause psychosocial dysfunctional traits to manifest in the child. Hence, if parents can cope effectively with the burden of care, the child will effectively adapt to the conditions of their illness.

### Strengths and limitations

This study addresses the existing research gap regarding the mental health of young children after liver transplantation. Further, it makes a significant and novel contribution in identifying that a lack of physical activity (< 30 min/day), poor quality of parent-child interactions, and parental mental health problems are risk factors for emotional and behavioral disorders in pediatric transplant patients. The results of this study indicate that further studies aimed at the developing evidence-based parenting interventions may be helpful in improving the long-term QOL of transplant patients. Additionally, this was a large sample retrospective research study, using high quality control group data by the PSM method that increased between-group comparability.

The limitations of this study also need to be considered. First, although large-sample data were used in this study, it was not obtained by a strict stratified sampling method. This limits the generalizability of the research. Second, this is a retrospective study which only provides evidence of the correlations between mental health of pediatric transplant patients and associated risk factors, and thus well-designed randomized controlled trials are needed to elucidate the causal relationship. Finally, the subjective measures used in the current study might be inevitably influenced by recalling bias.

## Conclusion

In conclusion, our data demonstrates a higher level of mental health problems among transplant patients aged 3 to 12 years, compared to the general population of healthy children. Further studies are needed to elucidate the impact of the childhood mental health of liver-transplant patients on their mental health in adulthood. Additionally, intervention studies may elucidate the effectiveness of the approaches for improving pediatric transplant patients’ mental health by increasing physical activity, facilitating adequate parent-child interactions, and offering treatment for parental mental health problems.

## Electronic supplementary material

Below is the link to the electronic supplementary material.

Supplementary Figure 1: Propensity score density of raw and matched groups.
Supplementary Figure 2: Distribution of propensity scores of matched treatment group and matched control group.

## Data Availability

Data sharing requests relating to data in this manuscript will be considered after the publication date. No end date exists for eligibility to submit a data sharing request for these data. Qualified researchers may submit a request containing the research objectives, the study/studies in scope, endpoints or outcomes of interest, statistical analysis plan, data requirements, publication plan, and qualifications of the researcher(s). A committee of internal advisors will review the requests. If not approved, a data sharing independent review panel will arbitrate and make the final decision. Upon approval, information necessary to address the research question will be provided under the terms of a data sharing agreement.

## Abbreviations

CPCIS: Chinese parent-child interaction scale
HRQOL: health related quality of life
ICCE: index of child care environment
PSM: propensity score matching
QOL: quality of life
SDQ: strengths and difficulties questionnaire
WHO-5: 5-Item World Health Organization Well-being Index
