# Supplementary material for: Mental health in children with living donor liver transplantation: a propensity score-matched analysis
Source: Child Adolesc Psychiatry Ment Health. 2022 Nov 29;16:94. doi: 10.1186/s13034-022-00516-4 (PMC9707263; doi:10.1186/s13034-022-00516-4)
Supplement: Supplementary file 1 — Supplementary Figure 1: Propensity score density of raw and matched groups. Supplementary Figure 2: Distribution of propensity scores of matched treatment group and matched control group. [file 13034_2022_516_MOESM1_ESM.docx]

**Figure Legends**

Supplementary Figure 1. Propensity score density of raw and matched groups.

Supplementary Figure 2. Distribution of propensity scores of matched treatment group and matched control group.


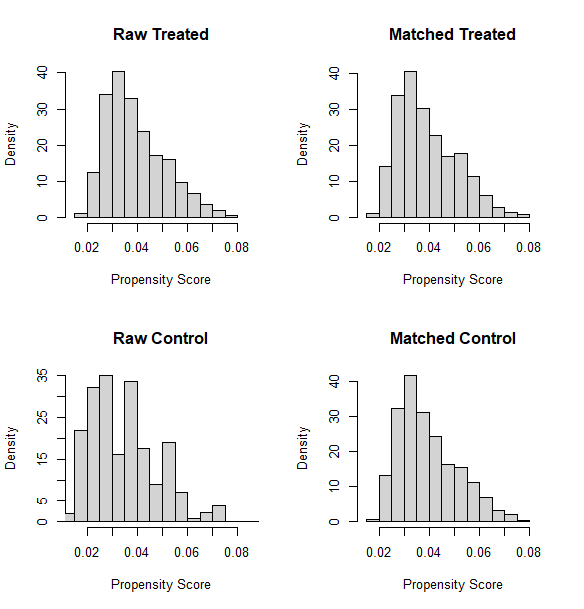


**Supplementary Figure 1. Propensity score density of raw and matched groups.**


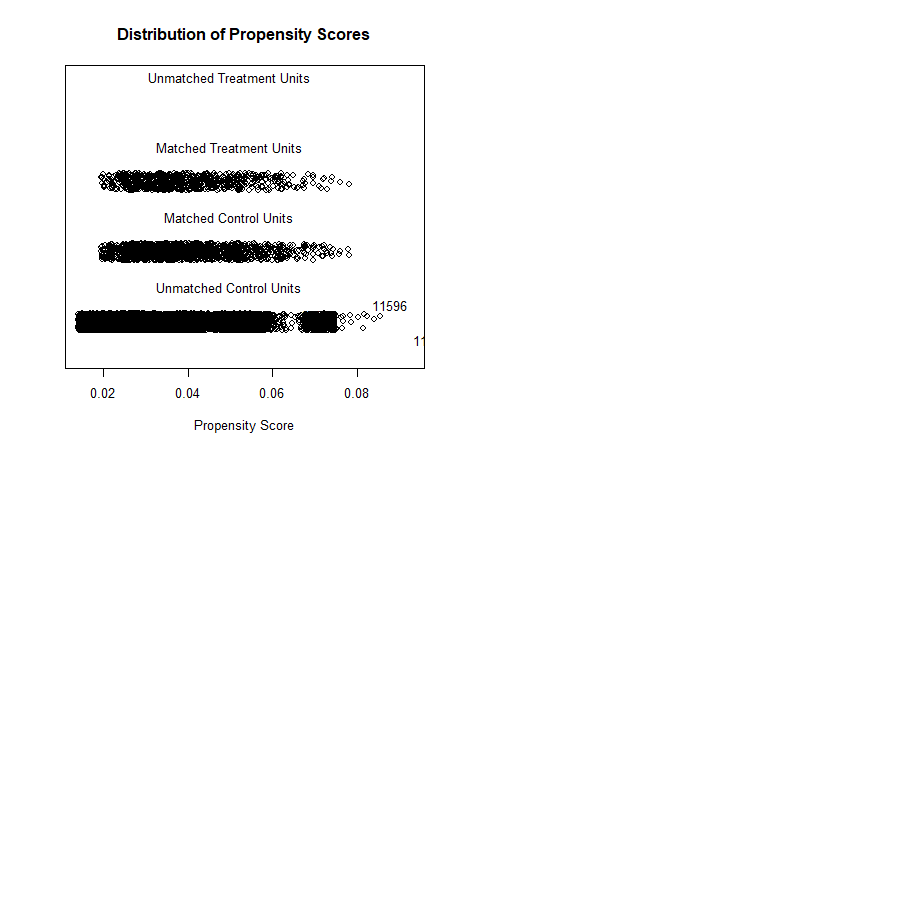


**Supplementary Figure 2. Distribution of propensity scores of matched treatment group and matched control group.**
